# IL-13Rα2 gene expression is a biomarker of adverse outcome in patients with adrenocortical carcinoma

**DOI:** 10.1371/journal.pone.0246632

**Published:** 2021-02-16

**Authors:** Abhinav Kumar, Ian H. Bellayr, Hridaya S. Singh, Raj K. Puri

**Affiliations:** 1 Division of Cellular and Gene Therapies, Tumor Vaccines and Biotechnology Branch, Center for Biologics and Evaluation Research, US Food and Drug Administration, Silver Spring, Maryland, United States of America; 2 Department of Zoology, Chaudhary Charan Singh University, Meerut, Uttar Pradesh, India; University of Nebraska Medical Center, UNITED STATES

## Abstract

Adrenocortical carcinoma (ACC) is a rare but aggressive endocrine malignancy that usually results in a fatal outcome. To allow the better clinical management and reduce mortality, we searched for clinical and molecular markers that are reliable predictor of disease severity and clinical outcome in ACC patients. We determined a correlation between the overexpression of *IL-13R*α2 and the clinical outcome in ACC patients using comprehensive data available in The Cancer Genome Atlas (TCGA) database. The dataset of 79 ACC subjects were divided into groups of low, medium, or high expression of *IL-13R*α2 as determined by RNA-seq. These patients were also stratified by length of survival, overall survival, incidence of a new tumor event, incidence of metastasis, and production of excess hormones. We report a correlation between *IL-13R*α2 expression and survival of subjects with ACC. High expression of *IL-13Rα*2 in ACC tumors was significantly associated with a lower patient survival rate and period of survival compared to low expression (p = 0.0084). In addition, high *IL-13R*α2 expression was significantly associated with a higher incidence of new tumor events and excess hormone production compared to low or medium *IL-13R*α2 expression. Within the cohort of patients that produced excess hormone, elevated *IL-13R*α2 expression was significantly associated with a lower survival rate. Additionally, *IL-13R*α1 had a potential relationship between transcript level and ACC survival. Our results and promising antitumor activity in preclinical models and trials indicate that *IL-13R*α2 expression is an important prognostic biomarker of ACC disease outcome and a promising target for therapeutic treatment of ACC.

## Introduction

Adrenocortical carcinoma (ACC) is a highly aggressive malignancy that originates in the outer layer of the adrenal gland. While most tumors of the adrenal gland are benign and common with a prevalence of 1–10%, ACCs are rare and occur with an annual incidence of 0.7 to 2.0 cases per million individuals. Clinically, ACC can be broadly divided into four stages, stage I and II tumors are restricted to organs where surgical removal is the common treatment, while advanced ACC stages defined as III and IV are highly fatal [1].

Approximately half of all ACC cases are discovered due to excess adrenal hormone produced by the patient. In these cases, the risk of mortality is high as the tumor has grown significantly and metastasized. Therefore, early detection is needed to reduce the high mortality rate from ACC. Treatment often includes tumor resection, adrenolytic drug mitotane, and cytotoxic therapy, but these options often have only moderate success [2]. Additional treatment options are needed to reduce the high mortality from this disease. Genes that are uniquely overexpressed in ACC may be promising targets for prognosis, early detection and therapeutic targets that could help manage this difficult disease.

We have previously reported that *IL-13R*α2 is overexpressed in several types of cancer including renal cell carcinoma [3], glioblastoma multiforme [4], ovarian cancer [5], colorectal cancer [6] and pancreatic cancer [7]. We have also reported that *IL-13R*α2 mRNA and protein is overexpressed in malignant ACC tumors compared to benign and normal samples [8]. *IL-13R*α2 was found to influence cell division and invasion in ACC [8]. An earlier genome-wide gene expression profiling study of malignant and benign ACC tumors reported that *IL-13R*α2 gene was transcriptionally upregulated by 24-fold in malignant compared to benign ACC tumors and had an excellent diagnostic accuracy for distinguishing malignant from benign adrenocortical tumors [9].

IL-13Rα2 is a component of the IL-13 receptor complex that consists of IL-13Rα1, and IL-4Rα chains [10–12]. IL-13 binds to IL-13Rα1 chain with low affinity and then recruits IL-4Rα chain to form a high affinity receptor for signal transduction. On the other hand, IL-13 binds to IL-13Rα2 with high affinity and can mediate signal transduction through this chain in diseased fibroblasts and tumor cells [6, 11]. It has been reported that extracellular domain of IL-13Rα2 is cleaved and serves as a decoy receptor for IL-13. Because IL-13Rα2 binds IL-13 with higher affinity than IL-13Rα1 [13, 14], it thereby allows sequestration of the ligand away from IL-13Rα1 for IL-13 signaling. It is proposed that this sequestration can be an apoptosis escape mechanism for tumor cells induced by IL-13 [15]. Inhibition of apoptosis of tumor cells that selectively express IL-13Rα2 suggests that IL-13Rα2 may act as an oncogene [16].

We have explored the therapeutic potential of IL-13Rα2 and reported that it can be targeted with an immunotoxin consisting of IL-13 and *Pseudomonas* exotoxin (IL-13-PE38QQR). We have tested this molecule in various Phase 1 and 2 clinical trials in patients with glioma and renal cell carcinoma [17]. Based on the overexpression of IL-13Rα2 in ACC tumors, we have performed a Phase 1 study in subjects with ACC [18]. Our results identified a maximum tolerated dose (MTD) and recommended further testing of this molecule at MTD in additional patients with ACC [18].

In this study, using a sizable dataset, we have examined whether the *IL-13Rα2* gene is associated with prognosis in ACC patients. We analyzed *IL-13Rα2* gene expression in patients with different clinical parameters. We accessed the National Cancer Institute’s (NCI) database, The Cancer Genome Atlas (TCGA), and analyzed for the overexpression of *IL-13R*α2 in ACC in RNAseq datasets. Related genes that are involved in the formation of *IL-13R* complex (*IL-13R*α1 and *IL-4R*) and program cell death ligand (*PD-L1)*, as a control, were also evaluated with respect to patient age, hormone production, new tumor events, and tumor metastasis. Our analysis identified a link between *IL-13R*α2 and the outcome of subjects with ACC. High expression of *IL-13Rα2* in ACC tumor samples resulted in a low survival rate and associated with tumor reoccurrence and excess hormone production.

## Materials and methods

### Dataset

The National Cancer Institute’s (NCI) TCGA database has collected a comprehensive dataset on clinical and genomic characterization of ACC patients through a network of physicians, scientists and bioinformatics experts. The available information includes the age, sex, racial background, clinical stage, and RNA sequence information from the tumor samples of individual ACC patients. We analyzed the RNAseq and corresponding clinical data files for 79 ACC patients which were downloaded in January 2017 from the TCGA Data portal. These datasets are now available from the Genomic Data Commons (GDC) Legacy archive. Protocols and procedural information for the data collection can be found at the GDC website. Additionally, GDC web applications were used in March 2017 to investigate mutations in ACC.

RNAseq data set was used to determine the *IL-13Ra2* transcript expression level in the tumors from the ACC patients. From RNASeqV2 platforms expression data was produced. MapSplice was used to do the alignment and RSEM software to perform the quantitation transcription expression abundance. Data was normalized using RPKM (Reads Per Kilobase of transcript, per Million mapped reads) and reference genome hg37 producing Level 3 expression data (https://www.ncbi.nlm.nih.gov/assembly/GCF_000001405.13/) [19].

For gene expression-based analyses, 79 ACC patients were stratified into three groups based on expression levels of the IL-13Rα2 transcript copy. The low expression group (n = 26) had an average IL-13Rα2 transcript copy of 4.19 ± 3.30 that ranged from 0.35–10.06 among the 26 patients. The medium expression group (n = 26) had an average IL-13Rα2 transcript copy of 47.78 ± 35.32 that ranged from 10.47–135.55 among the 26 patients. The high expression group (n = 27) had an average IL-13Rα2 transcript copy of 1310.28 ± 1603.21 that ranged from 143.98–7451.91 among the 27 patients. This strategy of dividing patients into three groups based on IL-13Rα2 transcript level within the tumor was used as the basis to evaluate any associations between the IL-13Rα2 expression level and disease progression and outcomes in ACC patients.

The GDC database also provides the genetic mutations in individual tumor gene sequences that exhibited altered expression in global transcriptional analysis in ACC tumors. However, analysis of dataset did not reveal any genetic mutations in the *IL-13Rα2* sequence in the 79 ACC tumors for which RNASeq data is available.

### Statistical analysis and software

Fishers exact test was used to show statistical significance (p ≤ 0.05) for categorical variables, such as patient survival, excess hormone production, new tumor events, and tumor metastasis using the Graph Pad suit of online tools (www.graphpad.com/quickcalcs/catMenu). The final survival was calculated at the endpoint (7 years) provided in TCGA dataset. We divided number of alive subjects by total number of subjects to determine the percentage of overall survival. Kaplan–Meier survival analysis was performed to compare patient survival between different *IL-13Ra2* expression levels using the Graph Pad Prism software. Primary data transformation and analysis was done through the JMP Genomics software suit (JMP Genomics 6.1). The GDC data portal and exploration web tools were used to acquire publicly available gene expression and mutational data in ACC (https://portal.gdc.cancer.gov/projects/TCGA-ACC).

## Results

### Patient characteristics, clinical information and disease outcome

Demographics data is summarized in Table 1. Among the 79 ACC subjects, 60.8% were female and 39.2% were male, the average age at diagnosis was 46 (range 14–83 years old) and the five-year survival rate was 65%. Gender and age at diagnosis did not have a significant effect on survival rate. Data pertaining to the clinical stage of ACC, incidence of a new tumor event, incidence of metastasis, and production of excess hormone were available for most of the subjects. Among the patient dataset, clinical stage classification was available for 77 of the 79 ACC subjects; 11.7% (9 subjects) had Stage I ACC and an 88% survival rate. 48.1% (37 subjects) had Stage II ACC and an 84% survival rate. 20.8% (16 subjects) had Stage III ACC and a 62.5% survival rate. 19.5% (15 subjects) had Stage IV ACC and a 26% survival rate. Of the subjects with excess hormone production, as classified by the TCGA, 16 expressed excess cortisol, 16 expressed excess cortisol and androgen, 8 expressed excess androgen, 3 expressed excess Mineralocorticoids, 2 expressed excess androgen and estrogen, 2 expressed excess estrogen, and 1 expressed excess cortisol and Mineralocorticoids.

**Table 1:** Demographic data.

Characteristics		N
Total		79
Sex	Male	31
	Female	48
Stage at Diagnosis	I	9
	II	37
	III	16
	IV	15
Metastatic Disease	Yes	17
	No	60
Sites of Metastasis	Liver	5
	Lung	5
	Multiple Sites	5
	Brain	1
	Lymph Node	1
Treatment Prior to Resection	Yes	0
	No	79
Adjuvant Treatment	Radiation	14
	Mitotane	43
Age at Diagnosis	Mean	46

Summary of demographic and clinical information on ACC subjects whose tumor samples were used to generate the transcriptional profiling data used in this study. ACC samples and clinical outcomes were collected from 79 subjects and deposited in the publicly available TCGA database. Metastatic and stage data for two subjects was unavailable.

Among the 79 ACC samples, clinical information regarding new tumor events was accessible for 73. Among these 73 subjects, the incidence of a new tumor event, defined as reoccurrence after initial treatment, was 47.9% (35 subjects). Subjects with a new tumor event had a 42% survival rate compared to 94% survival of subjects with no new tumor event. Among 77 ACC subjects, the incidence of metastasis was 22.1% (17 subjects). Subjects with metastatic tumors had a 29% survival rate (compared to 80% survival of subjects with non-metastatic tumors) and metastasis occurred in the lung (5 subjects), liver (5 subjects), brain (1 subject), lymph node (1 subject), or multiple sites (5 subjects).

Data available for 74 ACC subjects, 48 (64.9%) produced excess hormone. Subjects that produced excess hormone had a 56% survival rate compared to 88% survival of subjects that did not produce excess hormone. Hormones produced in excess included cortisol (16 subjects), cortisol/androgen (16 subjects), androgen (8 subjects), androgen/estrogen (2 subjects), estrogen (2 subjects), mineralocorticoids (3 subjects), and cortisol/mineralocorticoids (1 subject).

### Comparison of *IL-13R*α2 expression and ACC survival

The major objective of this study was to investigate the association of IL-13 receptor complex genes expression with disease outcome and hence could be used as potential biomarker and target for therapeutic treatment of ACC.

**Fig 1:**
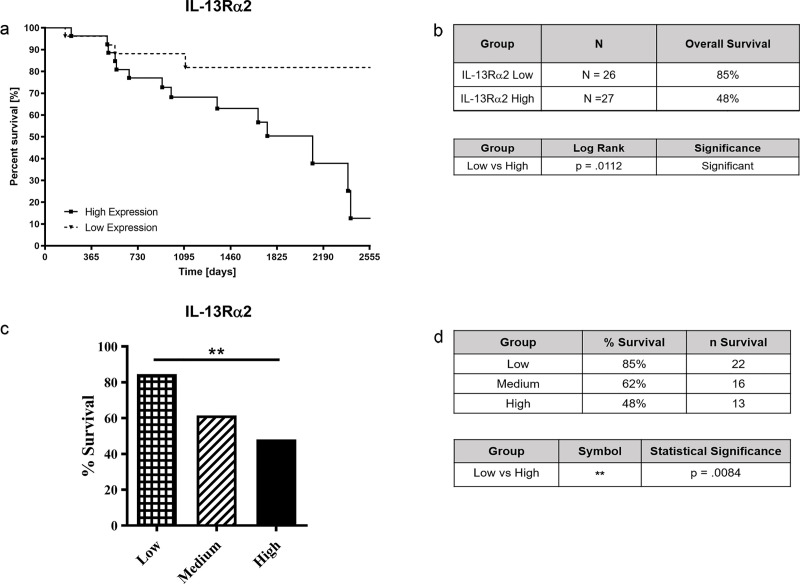
*IL-13Rα2* expression and survival analysis of patients with ACC. 79 ACC patients were divided between high (n = 27), medium (n = 26), and low (n = 26) *IL-13Rα2* expression and Kaplan-Meier survival analysis were performed to determine overall survival time over 7-year period (Fig 1A and 1B) and survival rate (Fig 1C and 1D). Median survival and confidence intervals were not provided at the TCGA database. Due to medium expression of IL-13Ra2 showing no statistical significance when compared with low or high expression, moderate expression group was not included in the figure. Data were analyzed for different *IL-13Rα2* expression levels and ACC survival using the Graph Pad Prism software. P values are shown comparing high vs. low expression.

We analyzed the gene expression in RNASeq dataset for a possible relationship between the expression of *IL-13Rα*2 gene and survival from ACC during the 7-year period (the maximum period for which patient follow up data is available in the database). Using global transcriptional profiles of tumors at the TCGA database, we divided the 79 ACC patients into groups of low (n = 26), medium (n = 26), or high (n = 27) expression of *IL-13Rα*2. To calculate the overall survival the number of alive subjects was divided by the total number of subjects. As shown in (Fig 1A), our analysis showed that the overexpression of *IL-13α2* in ACC tumors is associated with a lower patient survival time compared to low expression of *IL-13R*α2 (p = 0.0112) (Fig 1B). The moderate expression of IL-13Ra2 showed no statistical significance when compared with low or high expression groups.

High expression of the *IL-13R*α2 gene correlated with a lower rate of survival of ACC subjects. Subjects with low expression of *IL-13R*α2 had an 85% survival rate. In contrast, subjects with medium and high *IL-13R*α2 expression had a 62% and 48% survival rate, respectively (Fig 1C). Using the Fisher’s Exact Test, a statistically significant difference in the survival rate of subjects with low (n = 26) versus high (n = 27) expression of *IL-13R*α2 was observed (p = 0.0084) (Fig 1D). When comparing low vs medium (n = 26) and medium versus high expressions no statistically significant difference was found among groups.

### Comparison of *IL-13R*α2 expression and new tumor events

**Fig 2:**
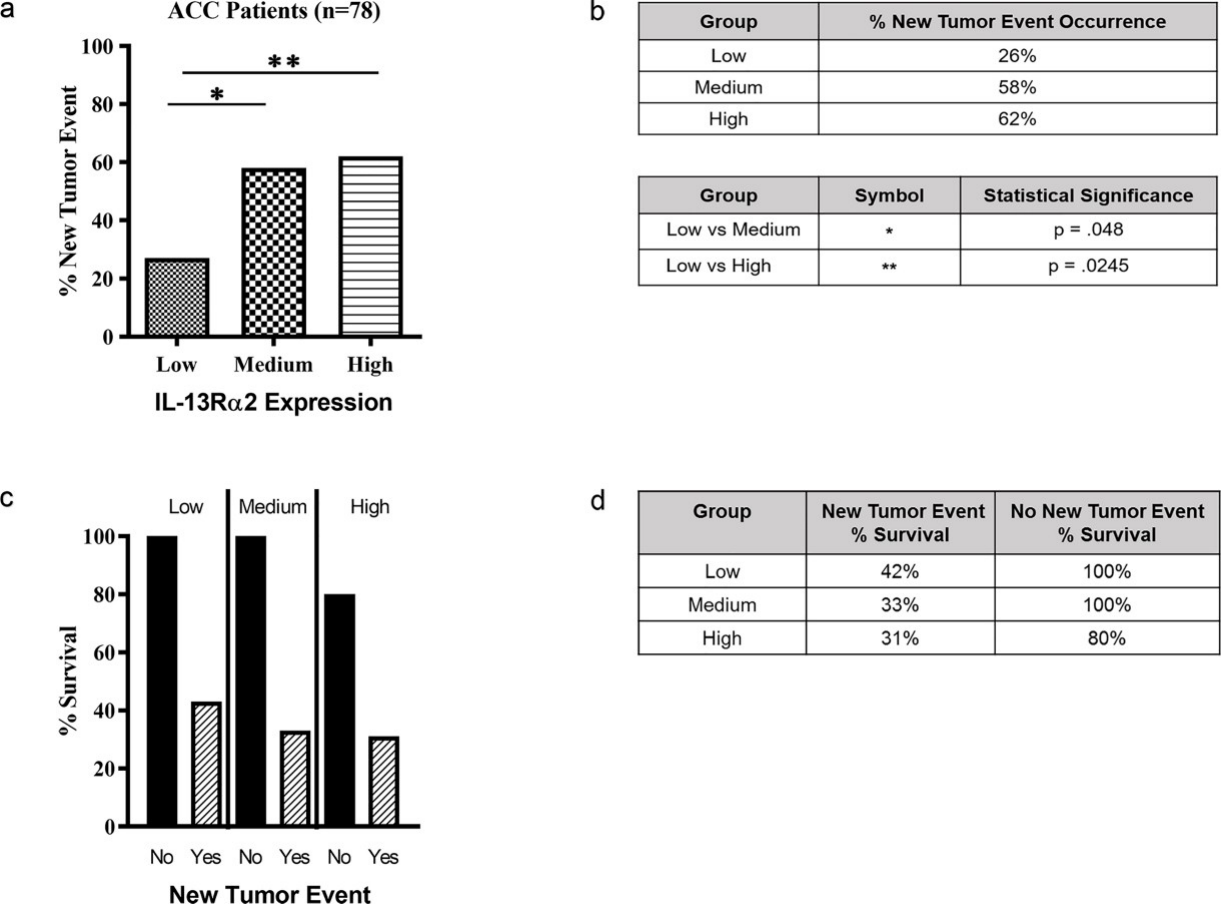
Comparison between *IL-13Rα2* expression levels and new tumor occurrence. 79 ACC subjects were divided between high (n = 27), medium (n = 26), and low (n = 26) *IL-13Rα2* expression and the new tumor occurrence in these expression groups (Fig 2A and 2B) and their relationship with survival in subjects who developed new tumor events and those without new tumor events (Fig 2C and 2D) was assessed. P values are shown where significant.

The frequency of a new tumor event, defined as reoccurrence after initial treatment, was 48% (38 subjects) among 78 ACC subjects (data for one subject was not provided). Subjects with a new tumor event had a 29% survival rate compared to a 95% survival rate of subjects with no new tumor event. A new tumor event occurred significantly more frequently in ACC subjects with medium (58%) (p = 0.0202) or high (62%) (p = 0.0042) *IL-13Ra2* expression compared to low (26%) *IL-13Ra2* expression (Fig 2A and 2B). However, among the 35 ACC patients with a new tumor event, the level of *IL-13R*α2 expression did not have a significant effect on the survival rate (Fig 2C and 2D).

### Comparison of *IL-13R*α2 expression and metastasis

**Fig 3:**
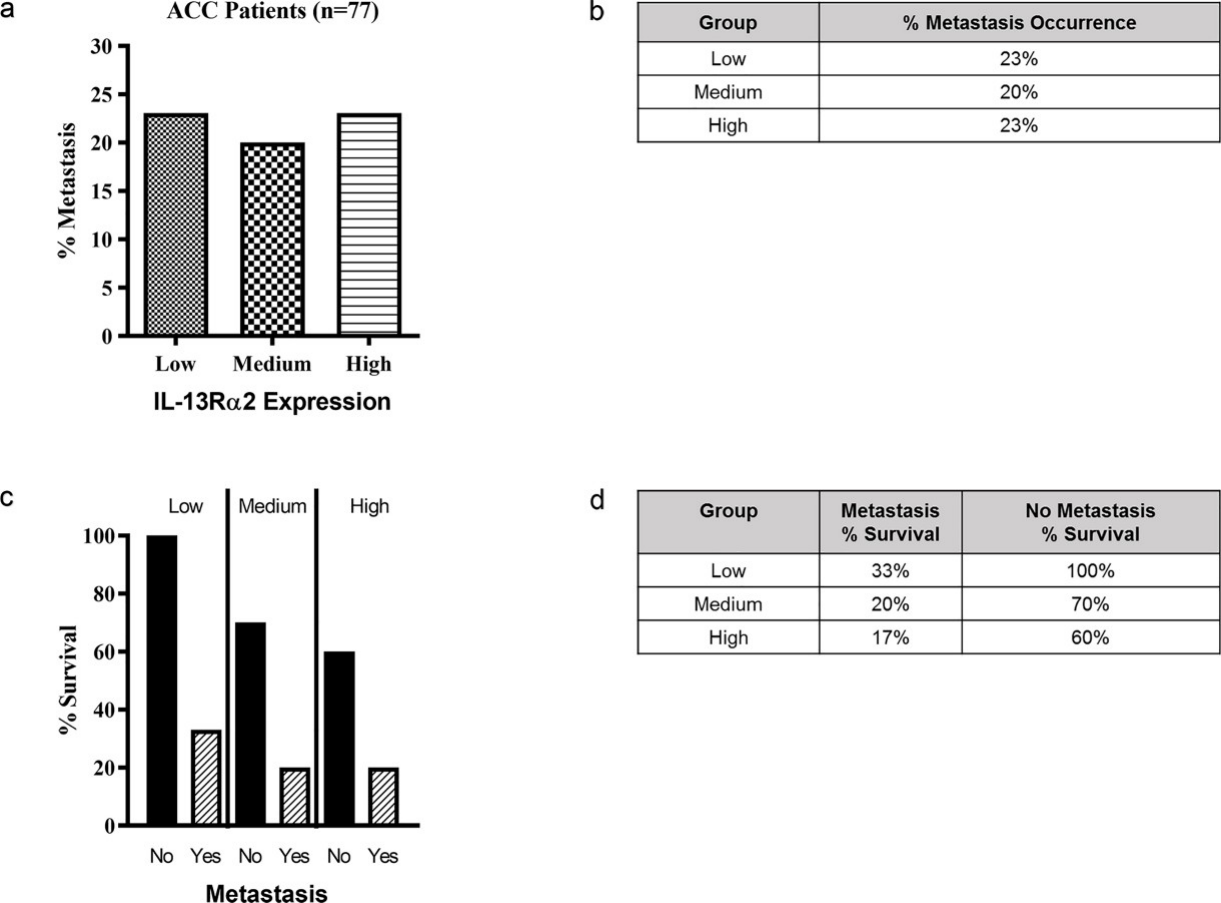
Comparison between *IL-13Rα2* expression levels and metastasis occurrence. 77 ACC subjects were divided between high (n = 27), medium (n = 26), and low (n = 26) *IL-13Rα2* expression and the metastasis occurrence in these expression groups (Fig 3A and 3B) and their relationship with survival in subjects who developed metastasis and those without metastasis was assessed (Fig 3C and 3D).

The incidence of metastasis was 22.1% (n = 17) among 77 ACC subjects and subjects with metastatic tumors had a significantly lower survival rate compared to subjects with non-metastatic tumors (24% versus 76% survival rate) (p = 0.0001). For *IL-13R*α2, there was no significant difference in the incidence of tumor metastasis among ACC subjects with low, medium, and high expression. Instead, metastasis occurred at an even rate between all three expression levels; the metastatic tumor incidence was 23% in ACC subjects with low *IL-13R*α2 expression, 20% in ACC subjects with medium *IL-13R*α2 expression, and 23% in ACC subjects with high *IL-13R*α2 expression (Fig 3A and 3B). Low *IL-13R*α2 expression (n = 6) was associated with a 33% survival rate of ACC subjects with tumor metastasis while ACC subjects without metastatic tumors (n = 20) showed a 100% survival rate (p = .001). In contrast, medium (n = 5) *IL-13R*α2 expression was associated with a 20% survival rate of ACC subjects with tumor metastasis while ACC subjects with medium (n = 20) *IL-13R*α2 expression and no metastasis showed a 70% survival rate (p = .1206). Similarly, high (n = 6) *IL-13R*α2 expression was associated with 16.7% survival rate of ACC subjects with tumor metastasis along with a survival rate of 60% for subjects without metastatic tumors (n = 20) (p = .1602). Thus, subjects with low *IL-13R*α2 expression showed statistically significant correlation between tumor metastasis and survival while this was not the case for medium and high expressors of *IL-13R*α2 (Fig 3C and 3D)

### Comparison of *IL-13R*α2 expression and production of excess hormone

**Fig 4:**
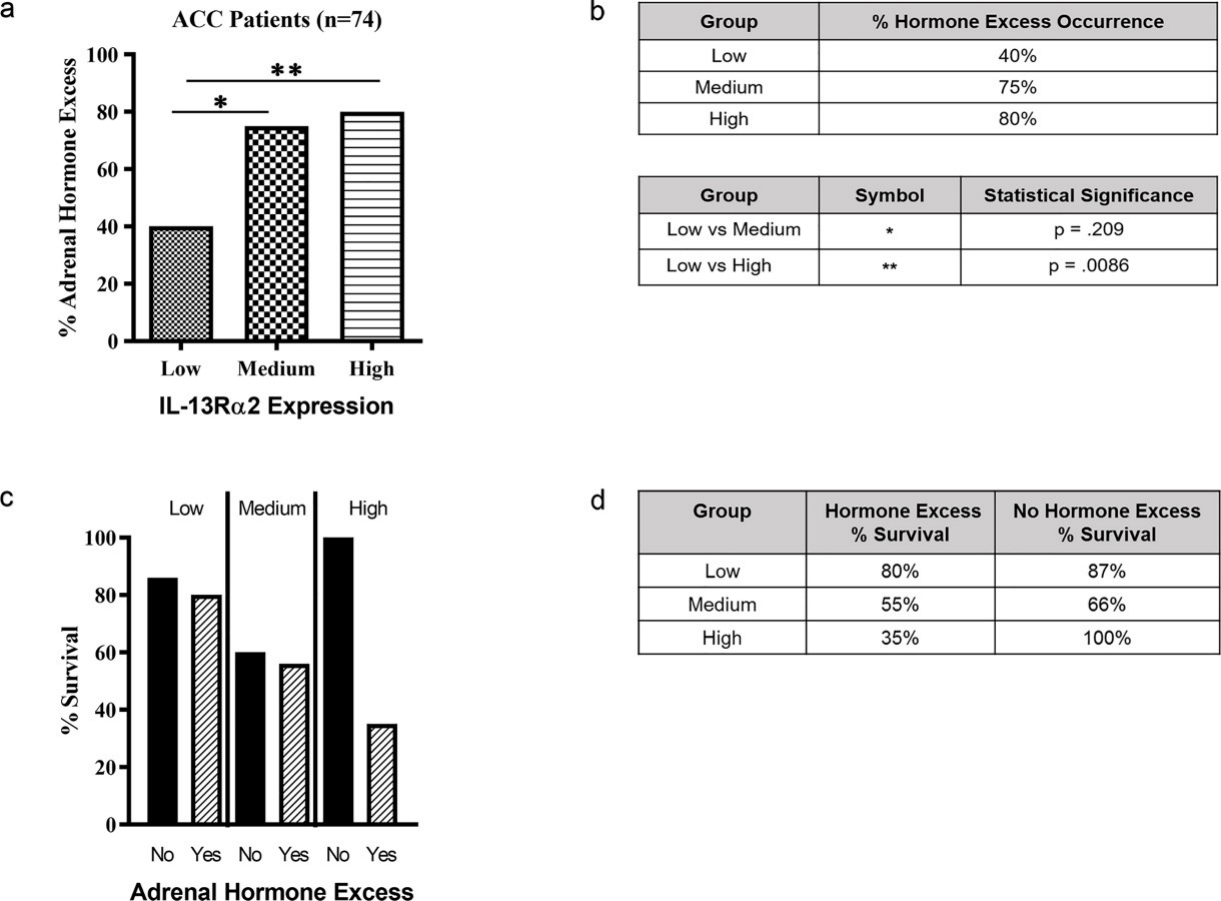
Comparison between *IL-13Rα2* expression and adrenal hormone excess. 74 ACC subjects were divided between high (n = 27), medium (n = 26), and low (n = 26) *IL-13Rα2* expression and their relationship with hormone excess occurrence was assessed (Fig 4A and 4B). P values are shown comparing low vs. medium and low vs. high *IL-13Rα2* expression. For comparison between *IL-13Rα2* expression, adrenal hormone excess and survival, and their correlation with survival in subjects who developed excess hormone and those without excess hormone was assessed (Fig 4C and 4D).

Among 79 ACC subjects, adrenal hormone excess data was available for 74 ACC subjects. In these subjects, 64.9% (48 subjects) produced excess hormone and subjects that produced excess hormone had a significantly lower survival rate compared to subjects that did not produce excess hormone (52% versus 85% survival rate) (p = 0.006). Interestingly, we found that excess hormone production occurred significantly less frequently in ACC subjects with low (40%) compared to medium (75%) or high (80%) expression of *IL-13R*α2 (p = 0.0209 for low versus medium and p = 0.0086 for low versus high) (Fig 4A and 4B). Although low *IL-13R*α2 was associated with an 80% survival rate whereas medium and high *IL-13R*α2 was associated with a 55% and 35% survival rate, respectively, these results were not statistically significant (Fig 4C and 4D).

### Analysis of *IL-13R*a1, *IL-4Ra* and PD-L1 expression and survival of subjects with ACC

**Fig 5:**
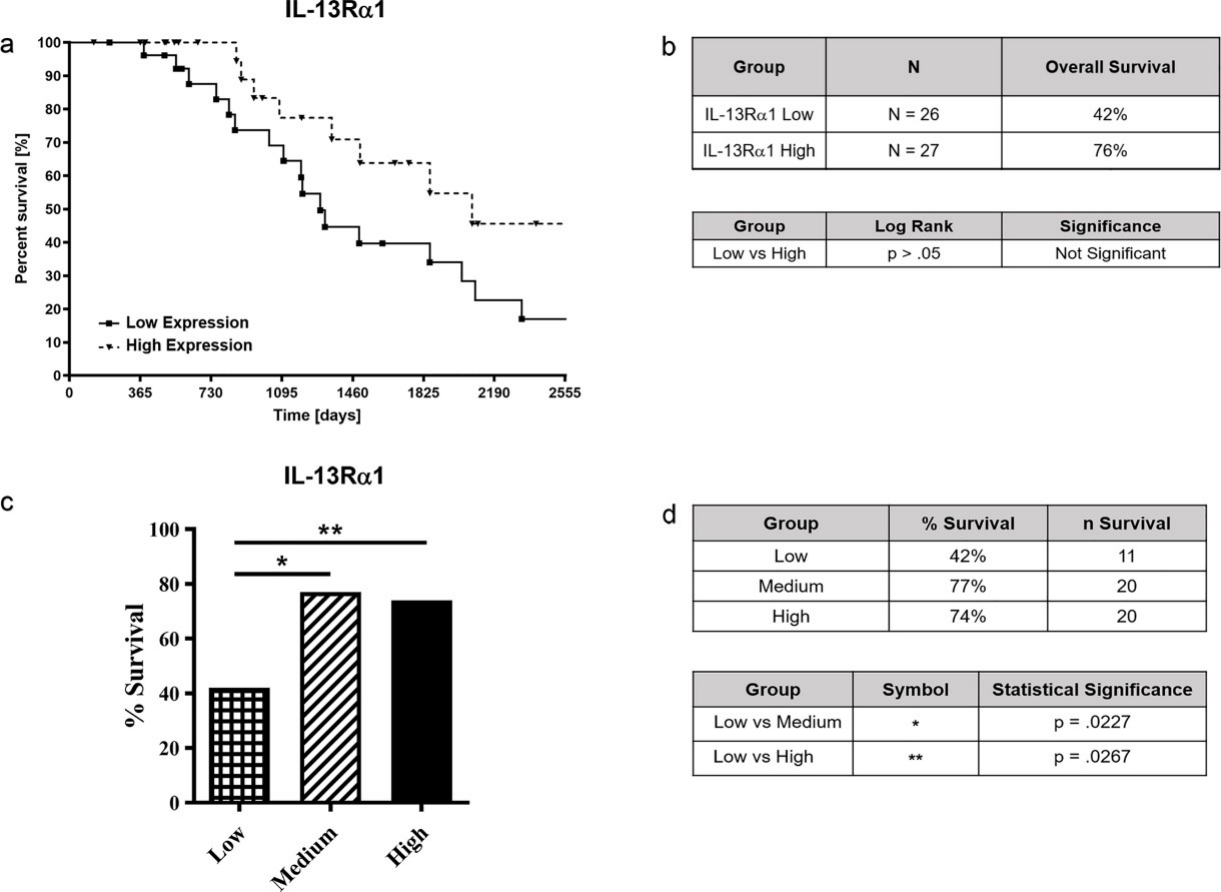
*IL-13Rα1* expression and survival analysis of patients with ACC. 79 ACC patients were divided between high (n = 27), medium (n = 26), and low (n = 26) *IL-13Rα1* (Fig 5A and 5B) expression and Kaplan-Meier survival analysis was performed to determine survival time over 7-year period. In addition, *IL-13Rα1* expression and its relationship with ACC overall survival was assessed (Fig 5C and 5D). Data was analyzed for different *IL-13Rα1* expression levels and ACC survival using the Graph Pad Prism software. P values are shown comparing high vs. low expression.

Since IL-13R and IL-4R constitute a part of IL-13 receptor complex, we also analyzed any possible association between IL-13Rα1 and IL-4Rα expression and ACC survival. In addition, we analyzed a relationship between PD-L1 expression and ACC survival. PD-L1 is a checkpoint inhibitor and shown to play a major role in suppressing T cell immunity during cancer. We divided the 79 ACC patients into groups of low (n = 26), medium (n = 26), or high (n = 27) transcription level of *IL-13R*α1. We observed a relationship between *IL-13R*α1 expression and ACC survival. Though the difference in length of survival was not statistically significant (p>0.05), our analysis showed that higher expression of *IL-13α1* in ACC tumors may be associated with a lower length of survival compared to low expression of *IL-13R*α1 (Fig 5A and 5B). In contrast with *IL-13Ra2* gene expression, subjects with low expression of *IL-13R*α1 had a 42% survival rate whereas subjects with medium and high *IL-13R*α1 expression had a 77% and 74% survival rate, respectively (Fig 5C and 5D). Using the Fisher’s Exact Test, there was a statistically significant difference in the overall survival rate of subjects with low (n = 26) versus medium (n = 26) (p = 0.0227) and low (n = 26) versus high (n = 27) (p = 0.0267) expression of *IL-13R*α1. Consistent with the observed relationship between *IL-13R*α1 expression and ACC survival, the age at death was also significantly higher in patients with medium (58.76 years) or high (62.33 year) versus low (34.76 years) *IL-13R*α1 expression. No statistical significance was observed between *IL-13R*α1 expression and the other clinical features measured from ACC patients. There was also no significant difference in the incidence of excess adrenal hormones among ACC subjects with low, medium, and high *IL-13R*a1 expression. Similarly, among the 48 ACC patients with excess adrenal hormones, the level of *IL-13R*α1 expression did not influence the survival rate. Additionally, there was no significant difference in the incidence of tumor metastasis among ACC subjects with low, medium, and high *IL-13R*a1 expression. Also, among the 17 ACC patients with tumor metastasis, the level of *IL-13R*α1 expression did not influence the survival rate. However, new tumor events occurred significantly more frequently in ACC subjects with low (64%) versus medium (34.6%) *IL-13R*α1 expression (p = 0.05). Importantly, among the 35 ACC patients with a new tumor event, the survival rate was significantly lower in patients with low (n = 16) versus elevated (medium and high) (n = 22) *IL-13R*α1 expression; patients with low *IL-13R*α1 expression had a 12.5% survival rate whereas patients with elevated *IL-13R*α1 expression had a 50% survival rate (p = 0.0356).

**Fig 6:**
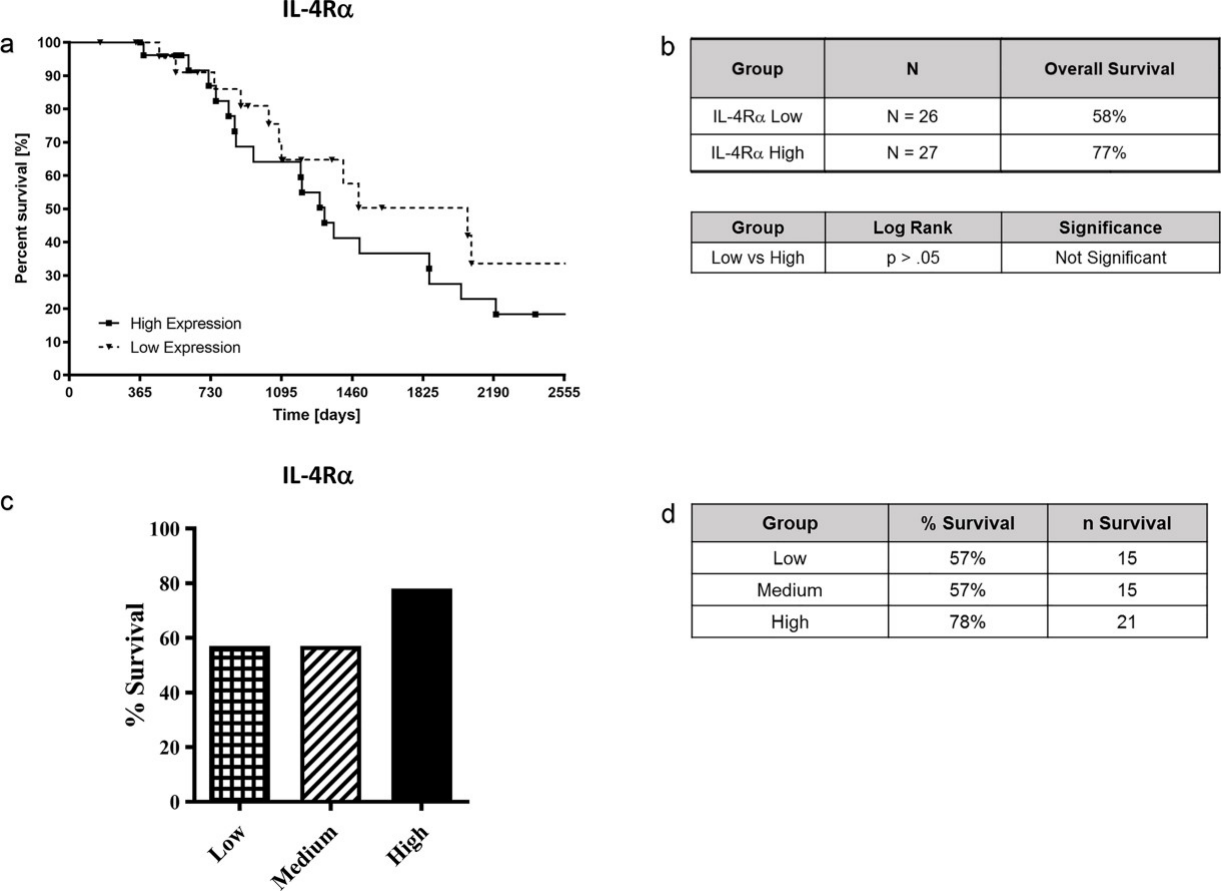
*IL-4Rα* expression and survival analysis of patients with ACC. 79 ACC patients were divided between high (n = 27), medium (n = 26), and low (n = 26) *IL-4Rα* expression and Kaplan-Meier survival analysis was performed to determine survival time over 7-year period (Fig 6A and 6B). In addition, *IL-4Rα* expression and its relationship with ACC survival was assessed (Fig 6C and 6D). Data was analyzed for different *IL-4Rα* expression levels and ACC survival using the Graph Pad Prism software. P values are shown comparing high vs. low expression.

**Fig 7:**
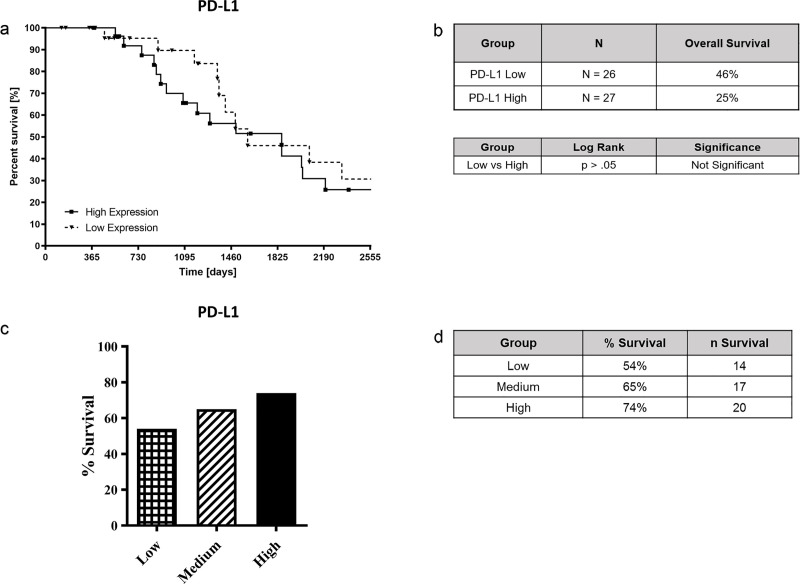
*PD-L1* expression and survival analysis of patients with ACC. 79 ACC patients were divided between high (n = 27), medium (n = 26), and low (n = 26) *PD-L1* expression and Kaplan-Meier survival analysis was performed to determine survival time over 7-year period (Fig 7A and 7B). In addition, *PD-L1* expression and its relationship with ACC survival was assessed (Fig 7C and 7D). Data was analyzed for different *PD-L1* expression levels and ACC survival using the Graph Pad Prism software. P values are shown comparing high vs. low expression.

79 ACC patients were also divided into groups of low (n = 26), medium (n = 26), or high (n = 27) for the *IL-4R*α and *PD-L1* expression. However, there was no statistical significance observed between the expression of *IL-4R*α and ACC survival (Fig 6). Similarly, *PD-L1* expression had no statistically significant relationship with survival in the 79 ACC subjects (Fig 7).

## Discussion

By analyzing the National Cancer Institute’s TCGA database, we demonstrated that IL13Rα2 gene expression is related with the survival of patients with ACC where analysis indicated that high *IL-13R*α2 expression is associated with negative clinical outcomes as measured by four different metrics. First, subjects with high *IL-13R*α2 expression had a lower survival rate and reduced length of survival than subjects with low *IL-13R*α2 expression. Second, subjects with medium and high *IL-13R*α2 expression had a higher incidence of a new tumor events than subjects with low *IL-13R*α2 expression. Third, subjects with medium and high *IL-13R*α2 expression exhibited a higher incidence of excess hormone production than subjects with low *IL-13R*α2 expression. Fourth, among subjects with excess hormone production, patients with high *IL-13R*α2 expression had a significantly lower survival rate compared to patients with low *IL-13R*α2 expression.

Our results illustrate a relationship between high *IL-13R*α2 expression and poor prognosis in patients diagnosed with ACC in agreement with our previous observations in patients with human glioblastoma multiforme (GBM) [4] which also utilized the NCI’s TCGA database. In previous studies, we analyzed *IL-13R*α2 expression by immunohistochemistry and RT-PCR in tumors derived from patients with ovarian [20] and head and neck cancer [21]. In both reports, we observed that high *IL-13Rα2* expression is associated with advanced stage disease. Collectively, these results indicate that *IL-13R*α2 could be a good prognostic biomarker in patients with tumors that express high levels of *IL-13R*α2.

The significance of *IL-13R*α2 expression in ACC tumors in not clear. It is possible that this receptor is mutated in ACC. Therefore, we searched the TCGA database for possible mutations in the sequence of the *IL-13R*α2 gene from ACC tumor samples. However, the TCGA database did not report any *IL-13R* genetic mutations in the *IL-13R*α2 gene suggesting that antigenic polymorphism did not contribute to the *IL-13R*α2 overexpression.

It is of interest to note that high expression of *IL-13R*α1 in ACC was associated with higher % of survival. This relationship was found in male patients only. Consistent with this observation, the age at death was also significantly higher in patients with high versus low *IL-13R*α1 expression. Another interesting finding was that low *IL-13R*α1 expression corresponds with new tumor events in ACC patients. In addition, we noted that survival from a new tumor event was related with an elevated *IL-13R*α1 expression.

Previous studies reported that *IL-13R*α1 can restrict tumor progression; IL-13 binding to *IL-13R*α1 activates Stat6 which promotes caspase-3 mediated apoptosis [15, 16, 22, 23]. Studies that have investigated the role of *IL-13Rα1* in cancer are conflicted. For example, Kwon *et al* showed that high expression of *IL-13R*α1 was associated with a lower risk of recurrence and cancer-induced mortality in patients with oral cavity squamous cell carcinoma [24]. In contrast, high *IL-13R*α1 expression was significantly associated with clinicopathological parameters of aggressive phenotypes and with reduced survival in patients with invasive breast cancer [25].

Because of varying observations of higher survival with high *IL-13R*a1 expression and contradictory relationships with other clinical parameters, we did not consider *IL-13R*α1 a clear prognostic biomarker. On the other hand, *IL-13R*a2 appears to be a reliable biomarker in ACC. The association between elevated *IL-13R*α2 gene expression and adverse clinical outcome suggests that measurement of *IL-13R*α2 in ACC patients could be used to differentially diagnose and identify patients at highest risk for a poor prognosis who could benefit from *IL-13R*α2 targeted therapy.

We also found that there was no significant correlation between transcriptional expression of *PD-L1* and ACC survival. However, new tumor events occurred significantly more frequently in ACC subjects with low (68%) compared to high (29%) (p = 0.0101) or medium and high (38%) (p = 0.0156) expression of *PD-L1*. Similar to our results, Fay *et al* reported that there was no relationship between *PD-L1* expression and ACC survival and clinic-pathologic parameters such as such as stage, grade, or excessive secretion of hormones [26].

Mitotane, which blocks hormone production, is administered in some ACC as treatment and shown to demonstrate overall 30% efficacy measured as stable disease or partial remission [2]. Interestingly, ribonucleotide reductase large subunit 1(RRM1) and cytochrome p450 2W1 (CYP2W1) expression levels has been associated with response to mitotane therapy and prolonged tumor-free survival [27, 28]. We investigated whether *IL-13R*α2 expression level can serve as a surrogate to monitor the efficacy of mitotane treatment in ACC patients. Analysis of the TCGA dataset revealed no statistical significance in survival between the low and high *IL-13R*α2 expressing ACC who had undergone mitotane treatment. However, these results should be interpreted with caution because of low sample size in the study sub-groups and the modest efficacy of mitotane against ACC.

Novel therapies are needed to increase the survival rate of ACC. Our results clearly indicate that patients with elevated levels of *IL-13R*α2 are at significantly higher risk of an adverse outcome. Therefore, a therapeutic treatment that targets *IL-13R*α2 may improve the prognosis and clinical outcome of subjects expressing elevated levels of *IL-13R*α2. Preclinical studies suggest that *IL-13R*α2 may be a promising therapeutic target for ACC. Studies have shown that IL-13-*Pseudomonas* exotoxin (IL-13-PE38QQR) is highly cytotoxic *in vitro* and *in vivo* to several types of *IL-13R*α2-positive cancer cells including ACC cells. Jain *et al*., demonstrated that the *IL-13R*α2-positive ACC cell line (NCI-H295R) is highly sensitive to IL-13-PE cytotoxin [8]. Furthermore, in this same study, it was shown that treatment of animals with IL-13-PE resulted in significant tumor regression and prolonged survival in a murine xenograft model of ACC. In addition, a Phase I clinical trial in patients with metastatic ACC demonstrated that IL-13-PE is safe and well tolerated and showed some activity in this disease. However, most patients developed neutralizing antibodies to immunotoxin which limited further administration of IL-13-PE. Immunodepletion prior to treatment is being considered in future clinical trials to improve the effectiveness of IL-13-PE as a therapeutic treatment for ACC [18].

In summary, our results clearly establish that the levels of *IL-13Rα2* gene expression play an important role in ACC pathogenesis and may serve as a prognostic biomarker of disease progression and adverse outcome in these patients. Additionally, mining of the TCGA datasets may allow development of *IL-13Rα2* gene detection-based test to guide decisions on case management, treatment options and monitoring the progress of ACC patients.
